# Maximum Incorporation of Soft Microgel at Interfaces of Water in Oil Emulsion Droplets Stabilized by Solid Silica Spheres

**DOI:** 10.3390/nano12152649

**Published:** 2022-08-01

**Authors:** Sebastian Stock, Susanne Röhl, Luca Mirau, Matthias Kraume, Regine von Klitzing

**Affiliations:** 1 Institute for Condensed Matter Physics, Technische Universität Darmstadt, 64289 Darmstadt, Germany; sebastian.stock@pkm.tu-darmstadt.de (S.S.); luca.mirau@pkm.tu-darmstadt.de (L.M.); 2 Department of Chemical and Process Engineering, Technische Universität Berlin, 10623 Berlin, Germany; s.roehl@tu-berlin.de (S.R.); matthias.kraume@tu-berlin.de (M.K.)

**Keywords:** microgels, pickering emulsions, simultaneous stabilization, coverage parameter

## Abstract

The incorporation of soft hydrophilic particles at the interface of water in non-polar oil emulsion droplets is crucial for several applications. However, the stabilization of water in non-polar oil emulsions with hydrophilic soft material alone is, besides certain exceptions, not possible. In our previous works, we showed that stabilizing the emulsions with well-characterized spherical hydrophobic silica nanospheres (SNs) and soft equally charged microgel particles (MGs) is a robust strategy to stabilize w/o emulsions while still incorporating a large amount of MGs at the interface. In the present study, we address the question of what the maximum amount of MGs at the interface in these kinds of emulsion droplets can be. By using well-characterized mono-disperse SNs, we are able to calculate the fraction of interface covered by the SNs and complementary that of the present MG. We found that it is not possible to decrease the SN coverage below 56% irrespective of MG softness and SN size. The findings elucidate new perspectives to the broader topic of soft/solid stabilized emulsions.

## 1. Introduction

The understanding of emulsions stabilized by soft or solid particles is essential in various fields of research and for even larger numbers of technical applications ranging from food science [1,2,3,4], medicine and pharmacy [5,6] to applications in the chemical industry [7,8]. The formation of particle stabilized emulsions, called Pickering emulsions (PEs), needs an energy input most commonly in the form of shaking, stirring or ultra-sonication to create the necessary interface where the particles can attach. In this context, *limited coalescence* describes the state of an emulsion after or during preparation when the droplets are formed and the initial strong coalescence process is ending due to the resistance of the forming particle barrier [9]. If the energy input of preparation is sufficiently high to produce more than enough interfacial space for all the particles, all particles will spontaneously attach to the interface [10,11,12]. In this case, the final emulsion structure and the resulting droplet diameter can be predicted from the total amount of particles and their geometry. Using silica spheres allows the application of simple geometric relations to calculate the amount of interface covered by particles versus the total droplet interface, called the coverage parameter. PEs are categorized by their phase composition in oil in water (o/w) or water in oil (w/o) emulsions. Besides the initial phase composition, the particles’ affinity to one or the other liquid decides the resulting emulsion type. Hydrophilic particles cause the formation of o/w emulsions while hydrophobic particles cause the formation of w/o emulsions [13]. The w/o type is very important for a large number of applications, such as creams and lotions, food grade emulsions [14,15] or for continuous product separation in interfacial catalysis where the oil phase constitutes most often the substrate and product phase [11,16]. In these cases, the presence of a certain type of hydrophilic soft particles at the interface is often essential or useful, e.g., a hydrophilic drug release system in case of medical creams [5,6] or a hydrophilic catalyst in the case of interfacial catalysis [11]. An example are microgel particles (MGs). They are able to act as both a protective drug encapsulation environment for hydrophilic substances [16,17,18] and an environmentally responsive drug release system [19,20,21,22]. MGs are cross-linked polymeric particles within the size range of about 1 nm to 1 µm. The use of MGs as stabilizers of emulsions bears serious inherent benefits. For instance, the use of whey protein MGs in the food industry may enable new healthier formulations for food grade emulsions [23]. In other applications, MGs may act as drug or catalyst delivery vehicles to the interface [16], and ultimately their responsivity can enable emulsion breakage on demand [24]. However, a drawback of their application is that most of the common MGs fall under the aforementioned category of hydrophilic particles and are not able to stabilize w/o emulsions on their own [15,25]. A solution to still ensure the formation of w/o emulsions is to deploy a second stabilizing agent. Jiang et al. [16], for instance, used hydrophilic fumed silica in combination with MGs to ensure the formation of water in toluene emulsions. The MGs were able to drag the catalyst to the interface, which improved the reaction performance.

In our previous work [26], we investigated the co-stabilization of w/o emulsions with positively charged poly-*N*-isopropylacrylamide (PNIPAM) MGs and likely charged silica nanospheres (SNs). These MGs are swollen or shrunken by water in response to a change in temperature. This behavior is called *volume phase transition* (VPT), and the temperature at which this process occurs is called *volume phase transition temperature* (VPTT, ∼32 °C for PNIPAM) [27]. This ability is rooted in the interaction between the solvent and the MG. The NIPAM monomer is prone to hydrogen bonding below the VPTT and loses this ability above, which causes temperature-induced swelling and shrinking. By investigating the emulsion stabilization by SNs and MGs in combination, we found that the formation of w/o emulsions stabilized simultaneously by SNs and MGs is possible, which was not using only MGs. In more detail, the MGs occupy their own space at the interface between the particles, resulting in a reduced droplet size of the MG/SN stabilized emulsions compared to SN-only stabilized emulsions. This work lead to the questions of what the maximum amount interfacial area is that can be covered by hydrophilic MG in between the hydrophobic solid particles when using a highly non-polar oil, and what governs it. We address these questions in the present work by varying the MGs’ cross-linker content, i.e., their softness, and the SN size. While keeping the amount of SN area content constant in each sample, we increase the amount of MG step-wise. By using well-characterized and modified SNs with detailed knowledge about their geometry, we are able to calculate how the SNs coverage parameter behaves with the increase in the present MGs. Vice versa, we can determine a maximum amount of MG that is incorporated in dependence on the tested system parameters. The applied MGs and SNs may be understood as a model system, and the found relations and the maximum droplet coverage of hydrophilic soft material may be extendable to other soft/solid co-stabilized emulsions.

## 2. Materials and Methods

### 2.1. Materials

Water with a specific resistance of 18.2 MΩ·cm at 25 °C was used from a Milli-Q purification system (Merck KGaA, Darmstadt, Germany). Ludox TM40 colloidal silica spheres, ethanol (>99%), 1-dodecene (>96%), dimethyloctadecyl[3-(trimethoxysilyl)propyl]ammonium chloride (60% in methanol), *N*-isopropylacrylamide (NIPAM) and *N*,*N*${}^{\prime }$-methylenbisacrylamid (BIS) were purchased from Sigma-Aldrich (Merck KGaA, Darmstadt, Germany). 2,2${}^{\prime }$-azobis-2-methyl-propanimidamide dihydrochloride (AAPH) was purchased from Cayman Chemical Company (Cayman Chemical, Ann Arbor, MI, USA).

### 2.2. Silica Nanosphere Preparation

The pristine silica nanospheres stem from two different sources. The smaller particles with a diameter of about 30 nm are commercially available (Ludox TM40). These particles were dialyzed for 10 days against about 50 L water. The larger particles with a diameter of about 100 nm were prepared from a sol gel process (Stoeber synthesis [28]) and were then grown with a seed growth approach in ethanol [29,30]. Both particle systems were surface-modified using dimethyloctadecyl[3-(trimethoxysilyl)propyl]ammonium chloride. For this, about 2 molecules of the silane per 1 nm^2^ of particle surface were added to the particle dispersion in ethanol, and the silanization was carried out for 1 h at room temperature and for 1 more hour at 60 °C. This modification hydrophobizes the particles and induces a positive surface charge. The Sauter mean diameter of the particles was determined by image analysis of TEM (FEI CM 20 ST, Philips, the Netherlands) micrographs of deposited SNs. Their average single particle density $\rho_{p}$ was evaluated from the total dispersion density of SN dispersions with varied concentration. For the density measurements, an oscillating u-tube densitometer (DM40, Mettler Toledo, Columbus, OH, USA) was used. For a more detailed description, see our previous publications [11,26].

### 2.3. Microgel Synthesis and Characterization

Microgel particles (MGs) were synthesized using a common and broadly studied recipe via precipitation polymerization reaction [27,31,32,33,34]. The monomer *N*-isopropylacrylamide (NIPAM) and the cross-linker *N*,*N*${}^{\prime }$-Methylenbisacrylamid (BIS) were dissolved in 120 mL water. The solution was degassed in a glass reactor under constant stirring (1000 RPM) and a constant nitrogen flow through the solution for at least 1 h at 80 °C. While the sum of NIPAM and BIS molecules was kept constant at 0.02 mol, their ratio to each other was varied to achieve different cross-linker densities. The cross-linker content is given as the molecular fraction of BIS to the total amount NIPAM and BIS molecules. Three different MG types were produced: lower cross-linked MG (2.5 mol% BIS), medium cross-linked MG (5 mol% BIS) and higher cross-linked MG (7.5 mol% BIS). The reaction was initiated by adding 33.5 mg 2,2${}^{\prime }$-azobis-2-methyl-propanimidamide dihydrochloride (AAPH, starter) in 1 mL water via a syringe and then carried out for 90 min at 80 °C and 1000 RPM. The obtained MGs were cleaned by dialysis for at least 10 days (10 cycles, 120 mL dispersion against 50 L water in total), dried by lyophilization and stored at −20 °C. The MG dispersions (0.006 wt%) were measured with dynamic light scattering (DLS) to obtain the hydrodynamic diameter with an DLS setup from LS instruments (Fribourg, Switzerland). The MGs ζ-potential was measured with a Zetasizer nano from Malvern Panalytical (Malvern, UK).

### 2.4. Emulsion Preparation

Prior to emulsion preparation, the stabilizing particles were dispersed in their preferred phase. The hydrophobic silica nanospheres were weighed in as a powder and dispersed using an ultrasonic bath for 10 min. The MGs were dispersed in the water phase using a multi-rotator (PTR-35, Grant-Bio, Grant Instruments, Shepreth, UK). The water phase was prepared by diluting the MG dispersion to the desired concentration and the water phase was poured to the oil phase (1-dodecene). All emulsion preparations were carried out at (8 ± 2) °C using an unsaturated ice-bath. The emulsions were mixed with an ultra turrax rotor stator mixer equipped with a S25N-10G dispersing unit for 5 min at 20 kRPM.

### 2.5. Microscopy and Drop Size Distribution

The emulsions were examined with light microscopy within about one hour after preparation with an Axio Imager A1 (Zeiss, Oberkochen, Germany). Microscopy was carried out by spreading a small volume of the emulsion of about 4 µL on a microscopy slide. For the droplet size distribution determination, at least 15 images from at least three different drops with in total a minimum of 300 droplets were imaged for each sample. The images were then analyzed using a commercially available droplet size determination software (SOPAT, Berlin, Germany).

### 2.6. Interfacial Tension Measurements via Pendant Drop Shape Analysis

The interfacial tension measurements were carried out at an inverse drop in a OCA 20 drop shape analyzer (DSA, Data Physics, Filderstadt, Germany). A cuvette (10 mm × 10 mm, 5 mL) was filled with the MG dispersion and a dodecene droplet of about (9 ± 0.2) µL was dispensed via a hook-shaped dispensing tip into the MG dispersion within a few seconds. The interfacial tension was measured every second for at least 30 min or until the drop ripped off. The averaged curves were calculated by averaging the measured values from at least 4 independent measurements, replacing the dispersion with a fresh one after two drops. The measurement error was taken from the standard deviation of this averaged values.

## 3. Results

### 3.1. Particle Characterization

The particles used for the stabilization of the emulsions are two types of solid silica nano-spheres (SNs) with a mean diameter of about 30 nm and 100 nm, respectively, and three types of soft hydrophilic MGs. The MGs were prepared using a lower (2.5 mol%), medium (5 mol%) and larger (7.5 mol%) amount of cross-linker. An overview of the related particle properties (size, charge, hydrophobicity) is given in Table 1 and Table 2. The SNs only differ in their size and are strongly similar regarding their positive surface charge and their hydrophobicity. The particles are hydrophobic with a contact angle (CA) slightly above 90°. In addition, the specific particle cross-section $a_{\emptyset }$ and the particle density $\rho_{P}$ were measured for a detailed analysis of the interfacial coverage.

**Figure 1:**
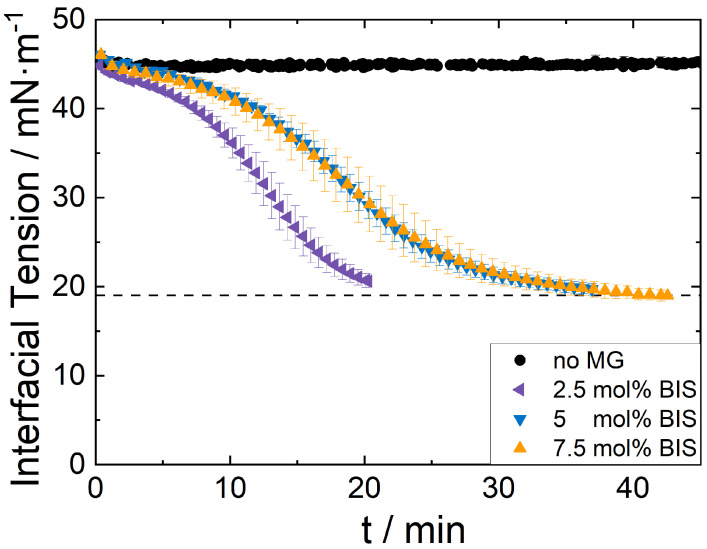
Interfacial tension of a dodecene droplet injected in an aqueous MG dispersion containing the three different MG types (cross-linker (BIS) content: low cross-linked MG: 2.5 mol%, medium cross-linked MG: 5 mol%, higher cross-linked 7.5 mol%), respectively, at a concentration of 0.0025 wt%. The interfacial tension decreases faster for the lower cross-linked MGs. The medium and higher cross-linked MG attach to the interface similarly fast.

The MGs were characterized regarding size and charge via dynamic light scattering and ζ-potential measurements. The size of the MGs was measured in repeated temperature cycles and averaged at temperatures below (20 °C) and above (50 °C) their VPTT of 32 °C. The results demonstrate the reversibility of their shrinking/swelling behavior. By averaging the size for high and low temperatures, respectively, the influence of hysteresis is captured in the form of the standard deviation. The measured hysteresis in the present case is with about less than 10% acceptably low. The size of all MGs is alike and shrinks slightly with increased cross-linker content. In contrast, their swellablity decreases strongly with the MGs cross-linker content. The stiffest MG exhibits a swelling ratio of only about *q* = 5.3, while the softer MG exhibits a swelling ratio of *q* = 19.9. This proves that with increased cross-linker density, the MGs become stiffer and less compressible as shown before in the literature [27,35,36]. Below VPTT, the MGs possess a lower ζ-potential, and their ζ-potential increases with the cross-linker content as usual [37,38]. In their shrunken state (above the VPTT) their charge is very similar for all cross-linker contents. The MGs’ differences in softness and those in their deformability have consequences for their ability to adsorb at interfaces. The time-resolved interfacial tension of a dodecene droplet in an MG solution was measured for all three MG types at low MG concentrations to evaluate their adsorption kinetics (Figure 1). At the same concentration, the decrease in interfacial tension is the fastest for the lower cross-linked MG. The medium and higher cross-linked MG exhibit a similar, slower kinetic. The final interfacial tension is very similar for all three MG types despite their differences in softness.

### 3.2. Droplet Size and Particle Coverage in PEs Stabilized by SNs Only

**Figure 2:**
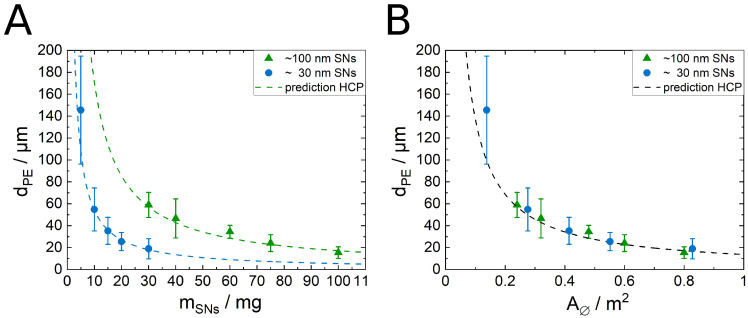
(**A**) Sauter mean diameter $d_{\mathrm{PE}}$ of emulsion droplets prepared with SNs only ($V_{\mathrm{PE},\mathrm{tot}}$ = 12.55 mL, 20 vol%, oil type 1-dodecene) as a function of the amount of SNs. The plot shows the typical reciprocal behavior of the particle mass. The dashed lines are completely independent predictions using Equation (1) and assuming a hcp (s=0.907). These predictions describe the data sufficiently (average deviation less than 10%). (**B**) Same measured Sauter mean droplet diameter as shown in (**A**) plotted over the total particle cross-section $A_{\emptyset }$ of the applied particles.

Using the SNs only to stabilize the emulsion leads to the typical reciprocal dependency of the (Sauter mean) droplet diameter $d_{\mathrm{PE}}$ from the SN mass $m_{\mathrm{SNs}}$. This behavior is described by Equation (1) [9,11,26]:

(1) $$d_{\mathrm{PE}}=\frac{6sf_{w}V_{\mathrm{PE},\mathrm{tot}}}{A_{\emptyset }}=\frac{6sf_{w}V_{\mathrm{PE},\mathrm{tot}}}{a_{\emptyset }}\frac{1}{m_{\mathrm{SNs}}}$$

where $f_{w}$ is the volume water fraction of the emulsion, $V_{\mathrm{PE},\mathrm{tot}}$ the total emulsion volume, $a_{\emptyset }$ the specific particle cross-section, *s* the coverage parameter and $A_{\emptyset }$ the total particle cross-section. The coverage parameter describes the relative amount of interface covered by the SNs per droplet area. Assuming that all particles are situated at the interface after the preparation, the 2D hexagonal close packing (hcp) is the maximum value of *s*. At 2D hcp the coverage parameter is s≈0.91. The measured data for the PEs stabilized solely by the larger and the smaller SNs, respectively, are shown in Figure 2. In all cases w/o emulsions are formed. This is confirmed by the tendency of the emulsions to settle down to the bottom of the vial and was shown in our previous works in detail [11,26]. The dashed lines in the plot are independent calculations using Equation (1), assuming a 2D hcp of the particles. These predictions are in very good agreement with the actually measured Sauter mean diameters of the prepared emulsions (average deviation less than 10%). Therefore, the assumption that all particles assemble at the interface during emulsion preparation is valid, and the data are in good agreement with previous studies on the same or similar particle systems [11,26]. When plotted over the total particle cross-section $A_{\emptyset }$ of the applied particles, the data can be described by a single curve.

### 3.3. Influence of Microgel Softness on the PE Structure

**Figure 3:**
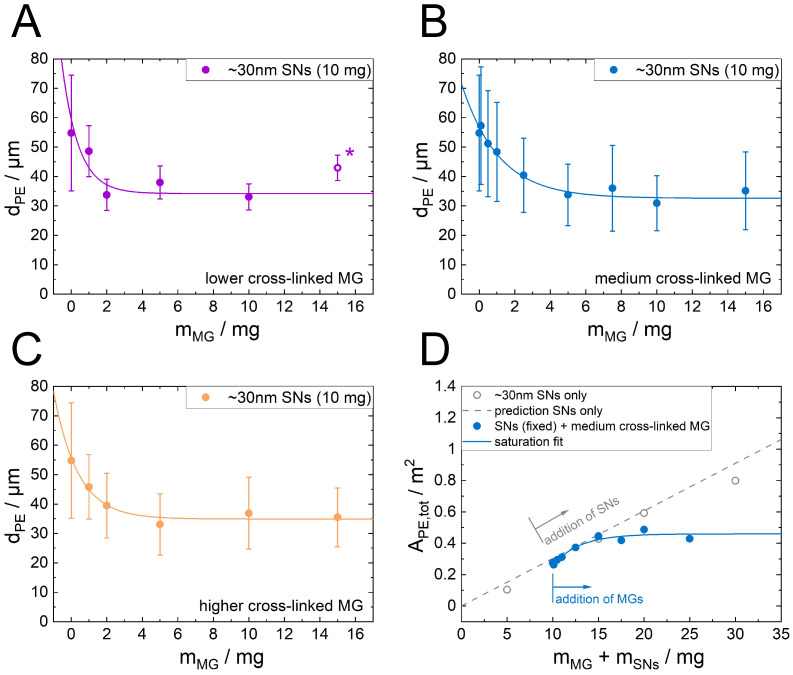
(**A**–**C**) Sauter mean diameter for emulsions ($V_{\mathrm{PE},\mathrm{tot}}$ = 12.55 mL, 20 vol%, oil type 1-dodecene) formed with a constant amount of SNs (10 mg) as a function of the MG content using MGs with a lower (**A**), medium (**B**) or higher (**C**) cross-linker content. The data are well described by Equation (2) (average deviation less than 9% for all fits). The emulsion formation with the highest amount of present MG with the lowest cross-linker content lead to the formation of a double emulsion with oil droplets inside the water phase. The data point marked with (*) was produced, evaluating only the outer droplet diameter while ignoring the inner droplets. The error bars represent the standard deviation of the droplet size distribution. (**D**) Total emulsion droplet area $A_{\mathrm{PE},\mathrm{tot}}$ for the emulsions prepared with the ∼30 nm SNs only and with the addition of the medium cross-linked MG over the total stabilizer mass calculated with Equation (3).

To test the interfaces capacity to take up and incorporate MG, emulsions with a constant amount of 10 mg SNs were formed while increasing the present amount of MGs. With that the total amount of stabilizer material increases. With the exception of the highest concentration of the lowest cross-linked MGs, w/o emulsions formed, clearly seen by the tendency of the emulsions to sediment and in agreement with earlier studies [26]. As shown in Figure 3A–C, the increasing amount of present MGs reduces the Sauter mean droplet diameter for all three different MG types.

**Figure 4:**
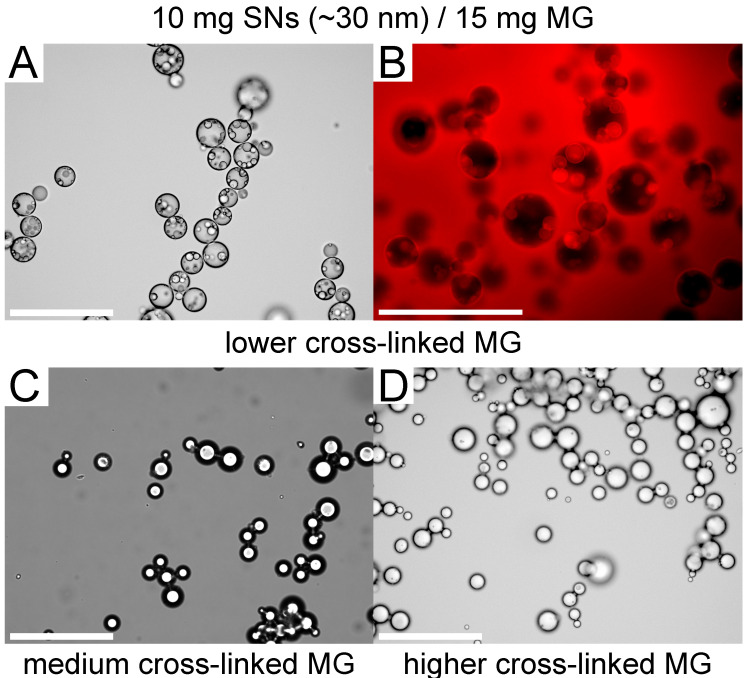
Micrographs of the emulsions with the highest amount of present MGs (15 mg). The o/w/o emulsion type for the emulsions prepared with the lowest cross-linked MG (**A**) was proven by dying the oil phase with nile red (**B**). For the higher crosslinked MGs (**C**,**D**), no double emulsions formed. The white bars represent 200 µm.

In all three cases, the curve levels at a plateau with increasing amount of present MGs. This limiting value is similar for all three MG types. In case of the lowest cross-linked MGs at the highest measured MG mass (15 mg), an oil in water in oil (o/w/o) double emulsion formed (Figure 4A,B), which was not the case for the other two higher cross-linked MGs at the same MG mass (Figure 4C,D).

By only measuring the outer droplet diameter and ignoring the oil droplets inside the water droplets, a droplet diameter roughly in the trend of the other MG concentration is found (marked with (*) in Figure 3A). This indicates that the inner droplets are mainly stabilized by the MGs. The saturation curves for the emulsions with constant SN mass and increasing MG mass are described by:

(2) $$d_{\mathrm{PE}}=d_{A}e^{-B_{d}m_{\mathrm{MG}}}+d_{\mathrm{limit}}$$

where $d_{A}$ is the amplitude and describes the maximum decrease in diameter by the MGs, $B_{d}$ describes the strength of the size loss per MG mass and $d_{\mathrm{limit}}$ is the final droplet size.

The total emulsion interface $A_{\mathrm{PE},\mathrm{tot}}$ can be calculated with the present volume of water ($V_{\mathrm{PE},\mathrm{tot}}\cdot f_{w}$) and the Sauter mean droplet diameter $d_{\mathrm{PE}}$:

(3) $$A_{\mathrm{PE},\mathrm{tot}}=\frac{6f_{w}V_{\mathrm{PE},\mathrm{tot}}}{d_{\mathrm{PE}}}$$

In the case of emulsions stabilized by the SNs, $A_{\mathrm{PE},\mathrm{tot}}$(SN only) can be predicted with the SN coverage parameter *s* and the incorporated amount of SN cross-section ($A_{\emptyset }=a_{\emptyset }\cdot m_{\mathrm{SNs}}$):

(4) $$A_{\mathrm{PE},\mathrm{tot}}=\frac{A_{\emptyset }}{s}=\frac{a_{\emptyset }\cdot m_{\mathrm{SNs}}}{s}$$

The results for $A_{\mathrm{PE},\mathrm{tot}}$ for the case of emulsion stabilization with the smaller SNs only and the addition of the medium cross-linked MG are shown in Figure 3D. In contrast to the SN only emulsions, which are predicted very well by Equation (4), the total emulsion area of the SN/MG emulsions does not scale linearly with the total stabilizer mass. It shows also a saturation behavior.

Under the assumption that even at the highest MG concentrations, all SNs are still present at the interface, the individual SN coverage *s* can be calculated by rearranging Equation (1). The rearrangement leads to Equation (5):

(5) $$s=\frac{a_{\emptyset }m_{\mathrm{SNs}}}{6f_{w}V_{\mathrm{PE},\mathrm{tot}}}d_{\mathrm{PE}}$$

**Figure 5:**
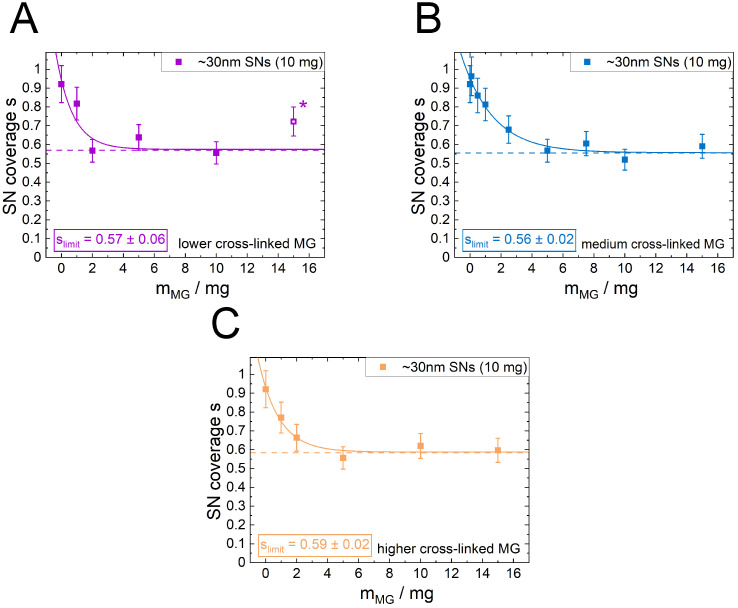
Coverage parameter *s* of the SNs in SN/MG co-stabilized emulsions calculated from the Sauter mean diameters presented in Figure 3A–C using Equation (5) (average deviation less than 8% for all fits). All three curves run into a saturation for high amounts of MGs. The final SN coverage $s_{\mathrm{limit}}$ was determined using Equation (6) and is very similar for all three MG types, irrespectively of the cross-linker content.

This linear transformation applied to the Sauter mean diameter curves from Figure 3A–C results in similar shaped curves for the SN coverage (Figure 5). The error bars were calculated from the propagation of uncertainty with the estimated uncertainty of $a_{\emptyset }$, which was assumed to be the largest insecurity. However, the absolute values for the initial and the final values of the coverage parameter *s* reveal further insights in the emulsion structure. For the PE samples without MGs, the received values are close to the 2D hcp value of 0.91. The final plateau value $s_{\mathrm{limit}}$ was determined using a simple saturation approach (Equation (6)) applied as a fit to the data:

(6) $$s=s_{A}e^{-B_{s}m_{\mathrm{MG}}}+s_{\mathrm{limit}}$$

In this empiric approach, $s_{A}$ is a value for the amplitude which is connected to the maximum amount of MG at the interface. $B_{s}$ is a value for the effectiveness of decreasing the drop size per MG mass and $s_{\mathrm{limit}}$ the desired value for the minimum percentage of covered interface by the SNs. This saturation approach describes the data sufficiently. The determined values for $s_{\mathrm{limit}}$ are between 0.56 and 0.59 for all three MG types. This shows that the cross-linker density has no significant influence on the final interface composition.

### 3.4. Influence of the SN Size on the PE Structure

**Figure 6:**
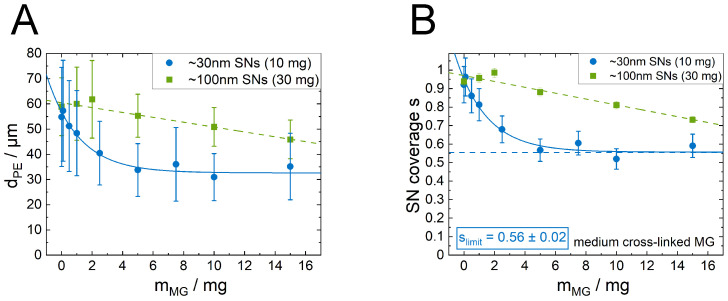
Comparison between emulsions stabilized with smaller and larger SNs, respectively, with increasing amount of the medium cross-linked MGs ($V_{\mathrm{PE},\mathrm{tot}}$ = 12.55 mL, 20 vol%, oil type 1-dodecene). The SN mass was adjusted so that both applied particle systems exhibit the same total particle cross section $A_{\emptyset }$. (**A**) The Sauter mean droplet diameter decreases in both cases with the present amount of MGs. A saturation behavior is only clearly visible in the case of the smaller SNs. (**B**) The calculations of the SN coverage parameter *s* shows that while the fraction of interface covered by the larger SNs decreases with the increasing present amount of MG, it never passes the minimum coverage parameters $s_{\mathrm{limit}}$ of around 0.56 calculated for the smaller SNs.

While the cross-linker content of the MGs has only minor effects on the behavior of the Sauter mean diameter over the increasing MG content, the size of the SNs changes this behavior significantly. For better comparison between smaller and the larger SNs, the amount of the larger SNs was adjusted so that their amount resembles a similar total particle cross section $A_{\emptyset }$ ($A_{\emptyset }=a_{\emptyset }\cdot m_{\mathrm{SNs}}$). Therefore, the experiments with the larger SNs were conducted with an SN mass of 30 mg. This leads to a starting point with a similar Sauter mean droplet diameter (compare the values for 10 mg 30 nm SNs and 30 mg 100 nm SNs in Figure 2). The determined Sauter mean droplet sizes with increasing amount of the medium cross-linked MGs are shown in Figure 6A in comparison to the data for the respective samples with the smaller SNs already shown in Figure 3B. The addition of MGs also decreases the droplet size when the larger SNs are present. However, the effect is less pronounced (decrease of 15 µm at 15 mg MG content) than for the PEs containing the smaller SNs (25 µm at 15 mg MG content). In contrast to the emulsions with the smaller SNs, the emulsions with the larger SNs do not show a clear saturation with MG, but the droplet sizes are in all cases larger than those of the PEs containing the smaller SNs. The saturation may therefore be better visible at higher MG contents. The calculation of the individual SN coverage *s* for every sample is shown in Figure 6B. Despite that this curve shows no clear saturation behavior, it shows that the SN coverage decreases from the hcp value with increasing MG content but always stays above the $s_{\mathrm{limit}}$ regime of around 0.56 determined for the smaller SNs. This indicates a saturation for larger MG content. The emulsions formed with the larger SNs show even more severe droplet clustering with high MG content, which prevents a reliable analysis of even higher MG contents.

## 4. Discussion

The SN characterization shows that they are very suitable model particles due to their well-defined shape differing only in size (Table 1). Both the MGs and the SNs are positively charged so that an aggregation of the two particle types is excluded. The MGs are roughly equally sized but differ strongly in their cross-linker density, resulting in differences of their swelling factors (Table 2). Low cross-linker density allows the MGs to shrink more than a higher cross-linker density. This means that the lower the MGs cross-linker density is, the softer the MGs are. At the interface, softer MGs are attached in a more flat state and are this way able to cover more interfacial area with the same amount of material [39]. This results in a faster decrease in the surface tension with increasing MG softness (Figure 1). The measured data are in good agreement with the studies of Tatry et al. [40] for the lower and medium cross-linked MG who also found an accelerated decrease in the surface tension for lower cross-linked MG. The adsorption kinetics are only accelerated for cross-linker densities <7.5 wt%. However, the final interfacial tension after long adsorption times is similar for all different cross-linker densities.

Stabilizing emulsions with the SNs only results in the typical reciprocal dependence of their Sauter mean diameter with applied amount of SNs. The fact that the completely independent predictions (dashed lines in Figure 2) calculated with Equation (1) are describing the data very well (average deviation less than 10%) shows that the assumptions for this geometrically derived formula are valid. These are (a) that all SNs are adsorbed at the interface and (b) they are packed at the interface in a 2D hexagonal pattern. Cryo-SEM studies in our and other previous studies did confirm this already [11,26]. The observation that the measured Sauter droplet diameters for both particle types are described by the same curve when plotted over the total particle cross-section $A_{\emptyset }$ (Figure 2B) shows that the applied particle area is the determining factor for the droplet size.

For the emulsions stabilized with MGs in addition, it was still assumed that all SNs are located at the interface after emulsion preparation. This assumption is reasonable, because the amount of interface created by the heavy stirring is high enough for all particles to potentially adsorb at the emerging droplet interface. The droplet size achieved by the stirring is given by the manufacturer of the stirrer as 1–10 µm. This corresponds to significantly more created interface than the interface of the droplets that is stabilized by the present interface active material which produces droplet sizes between 30 µm and 0 µm. The interfacial adsorption of the SNs is also backed up by cryo-SEM imaging in our previous paper [26]. With this technique, we could observe that the SNs are also able to adsorb onto the MGs but are preferable between them.

**Figure 7:**
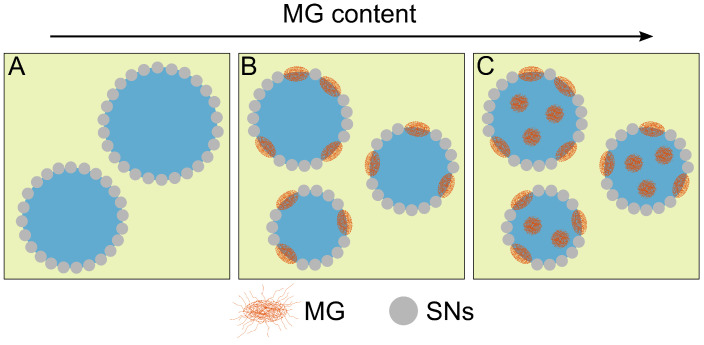
With increasing MG content, more MG is incorporated at the interface until saturation of the interface. (**A**) The emulsions stabilized by solid particles only show a tight packing of SNs at the interface. (**B**) The SN coverage is reduced with increasing amount of present MGs. (**C**) This incorporation is limited to a minimum SN coverage of around 0.56. Introducing more MG into the system only results in more MG situated in the bulk phase of the water droplets.

One key question now is to evaluate how the difference in the MGs’ softness and the difference in their adsorption kinetics influence the competitive adsorption against the SNs. Therefore, emulsions containing both particle types were prepared starting from emulsions with SNs only and increasing the amount of MGs. Instead of an expected reciprocal dependency of the Sauter mean diameter with the increasing amount of present stabilizing material, a saturation behavior was found (Figure 3A–C) irrespective of the softness of the MGs. Initially, when the interfacial capacity for the MGs is still very high, the droplet diameter decreases very strongly with the present amount of MG, i.e., a large proportion of the applied MG is incorporated at the interface. However, the size of the droplets remains constant at about 35 µm for a large amount of MGs in the system. This means that at a certain point, the increase in the present amount of MG is no longer decreasing the droplet size and with that is not increasing the total emulsion interface. This means vice versa that at a certain point the MG is rather located in the bulk water phase than adsorbing at the interface where only a maximum amount of MG is allowed. This mechanism is illustrated in Figure 7. This saturation is also visible in the data for the calculated total droplet area $A_{\mathrm{PE},\mathrm{tot}}$ (Figure 3D). The total droplet area does not increase linearly with the present total stabilizer mass. Therefore, a modification of Equation (5) or Equation (4) by including the potential covered area of the MGs additive to the SN coverage does not describe the situation well. Instead, a new saturation approach (Equation (2)) describes the data satisfactorily. This indicates that a stochastic process is present in the formation of the emulsions.

Due to the saturation of droplet size, the coverage of droplets by the SNs levels off at a constant value as well (Figure 5). The value of the minimum coverage parameter is around 0.56 for all applied MG systems, irrespectively of their softness. Neither the MGs softness nor their different adsorbing kinetics nor their minor differences in size and charge have an influence on the final amount of interface covered by the MGs. In detail, there may be a difference in material density at the locations where MGs are present at the interface, but this is not detectable by these simple geometric relations. Related to the constant droplet size, a maximum in MG coverage occurs as illustrated in Figure 7. These findings are in contrast to findings for o/w emulsion stabilization with MGs. Destribats et al. found that the softness of the MGs has a strong effect on the stability of the emulsions [41]. In their case a, lower cross-linker density, i.e., a high deformability, led to more stable emulsions. However, those findings are found in emulsion systems that are stabilized by MGs only. Therefore, these findings may not be transferable to the present case. Here, the explanation for the independence of the cross-linker density may be found in the coalescence phase during emulsion preparation. Droplets with a too-low amount of hydrophobic stabilizer at their interface are discriminated and coalesce until a sufficient amount of hydrophobic material is present. The MGs already attached to the interface in this process are buried under the increasing amount of SNs. In other words, the adsorption kinetics during the interface formation is overshadowed by the subsequent selection process in the early coalescence process on the way to the coalescence limit. This agrees with observations using cryo-SEM in our previous studies, where we could find SNs not only in between but also on top of the underlying MGs [26]. The found behavior that the softness of the MGs is irrelevant indicates once more that the MGs do not play a major role as a stabilizer for w/o but rather only occupy the interface.

The observation that double emulsions form for very high MG concentrations and lower cross-linker density of the MGs (i.e., a very high interfacial coverage potential) supports this point of view (Figure 4). It indicates that during stirring, a large variety of droplets of all types and interfacial compositions are formed, but only those with sufficient interfacial packing conditions survive the strong coalescence. In the case of very high MG content, the formed oil droplets in water may be small enough to be conserved inside the water droplets, which is only possible when a sufficient local MG interface coverage potential is present.

In retrospect, these observations clarify the emulsification behavior we observed in our previous study [26]. There, we kept constant the total stabilizer mass and varied the MG/SN ratio but always achieved the same droplet coverage of also s = 0.56. The explanation for this is that for all SN/MG ratios (15 mg/5 mg, 10 mg/10 mg, 5 mg/15 mg), the saturation of MG at the interface was achieved already. Vice versa, this observation from the previous study proves that the saturation behavior observed in the present study is independent of the SN concentration.

In contrast to small particles, for larger particles the SN coverage decreases linearly with increasing amount of MG (Figure 6) and no saturation occurs in the studied MG concentration regime. Nevertheless, the coverage parameter for the larger SNs also never falls below the minimum values determined for the smaller SNs. In opposite, the aim to incorporate as much MG as possible at the interface is easier to achieve when the SNs are smaller. A reason for the overall higher coverage parameter for larger SNs than for smaller SNs at the same MG concentrations is most likely attributed to their three times higher volume concentration to keep the total particle area constant. Thus, the adsorption probability during emulsion preparation is higher for the larger SNs than for the smaller ones when choosing the same $A_{\emptyset }$. This leads to a faster occupation speed of the larger SNs, reducing the free spots of the MGs.

## 5. Conclusions

In this work, we created a model system of monodisperse hydrophobic positively charged silica nano-spheres only differing in their size and positively charged hydrophilic MGs with different cross-linker density to modify their softness. With these well-characterized particle systems, we elaborate the question which is the maximum area that can be covered by hydrophilic MGs at the interface of w/o emulsions stabilized by the SNs when a highly non-polar oil is applied. We found that a minimum of about 56% of the interface needs to be covered by SNs, and a further increase in MG concentration does not increase the total emulsion interface further, irrespectively of the softness of the hydrophilic material. Only for larger silica spheres the found minimum SN coverage was higher. Thus, for the aim of including the most MG at the interface, smaller particles are better suited than larger ones. This knowledge is not only important for applications where formulations include MGs at emulsion interfaces, such as interfacial catalysis, but may also be considered as a model system for various applications where a mixture of soft and solid particles as cooperative interfacial stabilizers are applied.

## Figures and Tables

**Table 1:** Characterization of the applied SNs. The particles differ in size but are very similar regarding charge and hydrophobicity. ($d_{32}$ Sauter mean diameter of the particles (TEM), $\rho_{p}$ single particle density, $a_{\emptyset }$ specific particle cross-section, $a_{\mathrm{surface}}$ specific particle surface area, ζ zeta-potential in ethanol, CA contact angle against water).

	∼30 nm SNs	∼100 nm SNs
$d_{32}$/nm	27.6±3	97.0±5
$\rho_{p}$/g · cm^−3^	2.15±0.02	1.93±0.05
$a_{\emptyset }$/m^2^ · g^−1^	27.6±2.7	8.0±0.2
$a_{\mathrm{surface}}$/m^2^ · g^−1^	101.0±10.7	32.0±0.5
ζ/mV	+53±2	+53±4
CA/°	106±8	105±3

**Table 2:** Measured properties of the used MG systems.

	Lower Cross-Linked MG		Medium Cross-Linked MG		Higher Cross-Linked MG	
	20 °C	50 °C	20 °C	50 °C	20 °C	50 °C
$d_{H}$${}^{a}$/nm	736±21	272±34	616±25	269±35	611±9	351±3
*q*	19.9±7.7		12.1±4.9		5.3±0.3	
ζ${}^{a}$/mV	+5.7±1	+45.5±2	+15.3±2	+38.2±2	+19.9±2	+39.9±2

^a^ measured in water.

## Data Availability

The data presented in this study are available on request from the corresponding author.

## Abbreviations

The following abbreviations are used in this manuscript:

AAPH	2,2${}^{\prime }$-azobis-2-methyl-propanimidamide dihydrochloride
$A_{\emptyset }$	total particle cross-section
$a_{\emptyset }$	specific particle cross-section
$a_{\mathrm{surface}}$	specific particle surface area
$A_{PE,tot}$	total emulsion droplet interface
$B_{d}$	strength of size loss per MG mass
$B_{s}$	strength of silica nanosphere coverage loss per MG mass
BIS	*N*,*N*’-methylenbisacrylamid
CA	contact angle
$d_{32}$	Sauter mean particle diameter
$d_{A}$	maximum loss in droplet size
$d_{\mathrm{limit}}$	minimal droplet size for high MG concentrations
$d_{H}$	hydrodynamic microgel diameter
$d_{\mathrm{PE}}$	Sauter mean droplet diameter
$f_{w}$	volume water fraction
$m_{\mathrm{MG}}$	total microgel particle mass
MG	microgel particle
$m_{\mathrm{SNs}}$	total silica nanosphere mass
NIPAM	*N*-isopropylacrylamide
o/w	oil in water
PE	Pickering emulsion
PNIPAM	poly*N*-isopropylacrylamide
*q*	swelling ratio
$\rho_{p}$	average single particle density
*s*	silica nanosphere coverage
$s_{A}$	maximum loss in silica nanosphere coverage
$s_{\mathrm{limit}}$	final silica nanosphere coverage
SN	silica nanosphere
$V_{\mathrm{PE},\mathrm{tot}}$	total Pickering emulsion volume
VPT	volume phase transition
VPTT	volume phase transition temperature
w/o	water in oil
ζ	ζ-potential
